# Frozen Epitaxial Strain in Complex Oxide Freestanding Membranes

**DOI:** 10.1021/acsnano.6c08965

**Published:** 2026-10-05

**Authors:** Paul Lenharth, Yuchuan Yao, Shihao Tu, Pratap Pal, Varun Nitin Mapara, Ruiqi Sun, Wuzhang Fang, Peyton Burden, Tula R. Paudel, Jason Kawasaki, Uwe Bergmann, Hua Zhou, Yuan Ping, Chang-Beom Eom

**Affiliations:** † Department of Materials Science and Engineering, 5228 University of Wisconsin-Madison, Madison, Wisconsin 53706, United States; ‡ Department of Physics, University of Wisconsin–Madison, Madison, Wisconsin 53706, United States; § Department of Physics, 6806 South Dakota School of Mines and Technology, Rapid City, South Dakota 57701, United States; ∥ X-ray Science Division, Advanced Photon Source, 1291 Argonne National Laboratory, Lemont, Illinois 60439, United States

**Keywords:** epitaxial strain, freestanding membrane, X-ray diffraction, XPS, oxygen vacancy

## Abstract

Transition metal complex oxides exhibit a diverse array of emergent physical properties engineered through epitaxial strain. However, the interpretation of strain-driven phenomena is often obscured by structural modifications—particularly under in-plane tension—which can trigger unexpected departures from bulk behavior. Here, we report the observation of “frozen epitaxial strain” in complex oxide heterostructures, where epitaxial tensile strain induces a unit-cell volume expansion that remains locked even after complete substrate release. Using synchrotron X-ray diffraction, we quantify this irreversible structural response, demonstrating that the lattice fails to recover its bulk-like dimensions in the freestanding state. X-ray photoelectron spectroscopy and first-principles calculations reveal that this expansion is stabilized by a high density of tensile strain-promoted oxygen vacancies. These findings establish a fundamental link between epitaxial tension, defect chemistry, and structural “memory,” providing critical design rules for the controlled fabrication of functional freestanding membranes and strain-engineered oxide electronics.

## Graphical abstract

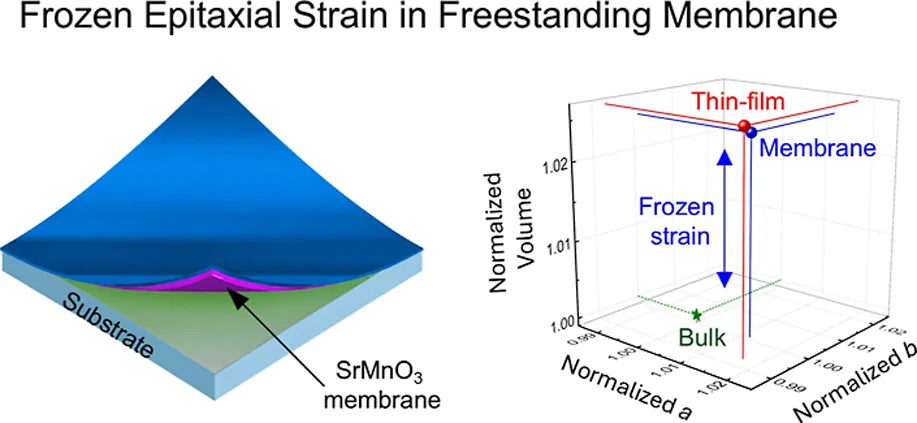

## Introduction

Transition metal complex oxides (ABO_3_) provide a powerful platform for engineering emergent physical properties through epitaxial strain-induced structural modifications.^1–5^ This strain-structure–property paradigm has led to a breadth of discoveries, ranging from strain-induced ferroelectricity^6,7^ and metal–insulator transitions^8^ to the formation of interfacial two-dimensional electron gases^9^ and emergent magnetism.^10^ However, a central challenge remains: epitaxial strain does not solely distort the lattice elastically, but can also alter stoichiometry by modifying defect formation energy.^11,12^ In particular, tensile biaxial strain frequently lowers the formation energy of oxygen vacancies,^13,14^ driving off-stoichiometry^15^ and unit-cell volume expansion that fundamentally reshapes charge, spin, and orbital configurations.^16^ Consequently, observed property changes often reflect an inseparable convolution of elastic strain and strain-induced defect chemistry.

In this context, freestanding oxide membranes^17–21^ offer a unique opportunity to decouple these intertwined contributions. Removal of the epitaxial substrate eliminates mechanical clamping, theoretically relieving elastic strain. If structural distortions persist after release, they must arise from “frozen strain” - a defect-mediated stabilization of the lattice during growth rather than purely mechanical deformation.

Here, we employ SrMnO_3_ as a model system to directly probe this phenomenon.^22–28^ SrMnO_3_ is a cubic G-type antiferromagnetic^29^ perovskite with lattice parameter close to commercially available substrates, enabling high-quality epitaxial thin-film growth under multiple controlled tensile strain states.^30–33^ Its electronic and magnetic phases are highly sensitive to biaxial strain,^31,34^ yet its strong tendency to form oxygen vacancies complicates the realization of intrinsic strain-driven phases.^7,22,28,30,35–37^ Previous experimental and theoretical studies have shown that tensile strain lowers the oxygen-vacancy formation energy in SrMnO_3_, leading to oxygen-deficient phases accompanied by lattice expansion, modifications of Mn valence, and substantial changes in the electronic and magnetic properties.^26,30,38–41^ These findings established a strong coupling between epitaxial strain and defect chemistry in SrMnO_3_, making it difficult to isolate the intrinsic effects of elastic lattice distortions in conventional substrate-clamped films. Freestanding oxide membranes provide an opportunity to disentangle these contributions by removing the epitaxial constraint while preserving the defect configurations established during growth, thereby enabling direct examination of whether defect-induced structural distortions persist after strain release. Toward this goal, by combining synchrotron X-ray diffraction with first-principles calculations, we demonstrate that tensile strain induces a unit-cell volume expansion that remains locked even after substrate removal. Our results establish a direct link between epitaxial tension, strain induced defect formation and irreversible structural memory, providing a new framework for designing membrane-based functional devices.

## Results and Discussions

### Growth of High-Quality Epitaxial Thin-Film and Freestanding Membranes

To investigate strain-induced structural evolution and its persistence after release, epitaxial SrMnO_3_ (SMO) thin films were grown on (001)-oriented lattice-mismatched (LaAlO_3_)_0.3_(Sr_2_AlTaO_6_)_0.7_ (LSAT) and SrTiO_3_ (STO) cubic substrates (Figure 1a) using pulsed laser deposition, following similar growth conditions established in previous studies of epitaxial SMO thin films.^42,43^ A ∼10 nm water-soluble SrCa_2_Al_2_O_6_ (SCAO) sacrificial layer (pseudocubic lattice parameter ≈ 3.859 Å), which is nearly lattice matched to LSAT substrate (3.868 Å),^4,44^ was grown to enable subsequent membrane fabrication (Figure 1a,b). The small lattice mismatch and limited SCAO thickness (∼10 nm) promote coherent epitaxial growth and minimize strain-relaxation effects prior to SMO deposition. A 2 nm STO barrier layer was inserted between the SCAO and SMO layers to suppress cation interdiffusion, thereby preserving the structural integrity and water solubility of the sacrificial layer.^43^ Throughout this paper, we have focused our discussion on the tensile-strained thin film (SrMnO_3__*F*) grown on LSAT substrate (imposes 1.66% in-plane tensile strain) and corresponding released membrane (SrMnO_3__*M*). Comparisons are drawn with higher tensile strain thin film (SrMnO_3_/STO_*F*) grown on STO substrate (imposes 2.6% in-plane tensile strain) and corresponding membrane (SrMnO_3_/STO_*M*). Detailed thin-film growth parameters and freestanding membrane processing steps (Figure S1) are provided in Methods section.

**Figure 1:**
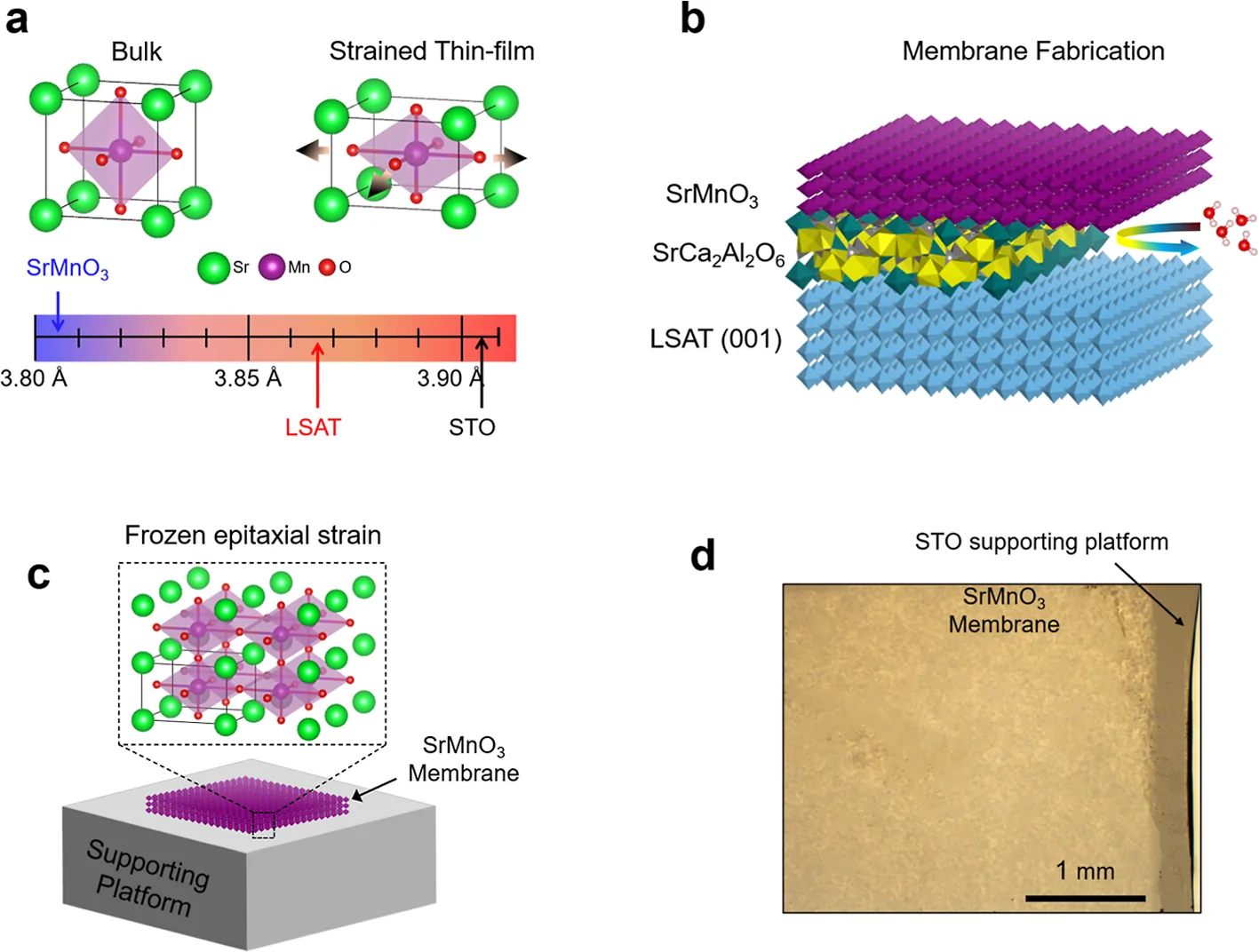
Fabrication and frozen epitaxial strain of freestanding SrMnO_3_ membrane. (a) Comparison of the lattice parameters of bulk SrMnO_3_, and the LSAT and STO substrates. Epitaxial growth on LSAT results in a biaxial tensile strain in the SrMnO_3_ film. (b) Schematic illustration of membrane fabrication via selective dissolution of the sacrificial layer. (c) Schematic of the transferred freestanding membrane after polymer removal. After release from the substrate, the membrane may retain frozen epitaxial strain information. (d) Optical image of a transferred membrane on STO support, derived from LSAT substrate. A nearly 5 mm × 5 mm membrane resulted from the fabrication process.

High crystallinity and epitaxial registry were confirmed by X-ray diffraction and reciprocal space mapping. Sharp film reflections with pronounced Laue oscillations (Figures 2a and S3a) indicate smooth interfaces and uniform thickness, while reciprocal space maps (RSM) around asymmetric reflections demonstrate fully coherent growth on both LSAT and STO substrates (Figures 2b and S3b). Atomic force microscopy reveals atomically smooth surfaces with clear terrace structures (Figure S5), confirming high structural quality prior to membrane release.

**Figure 2:**
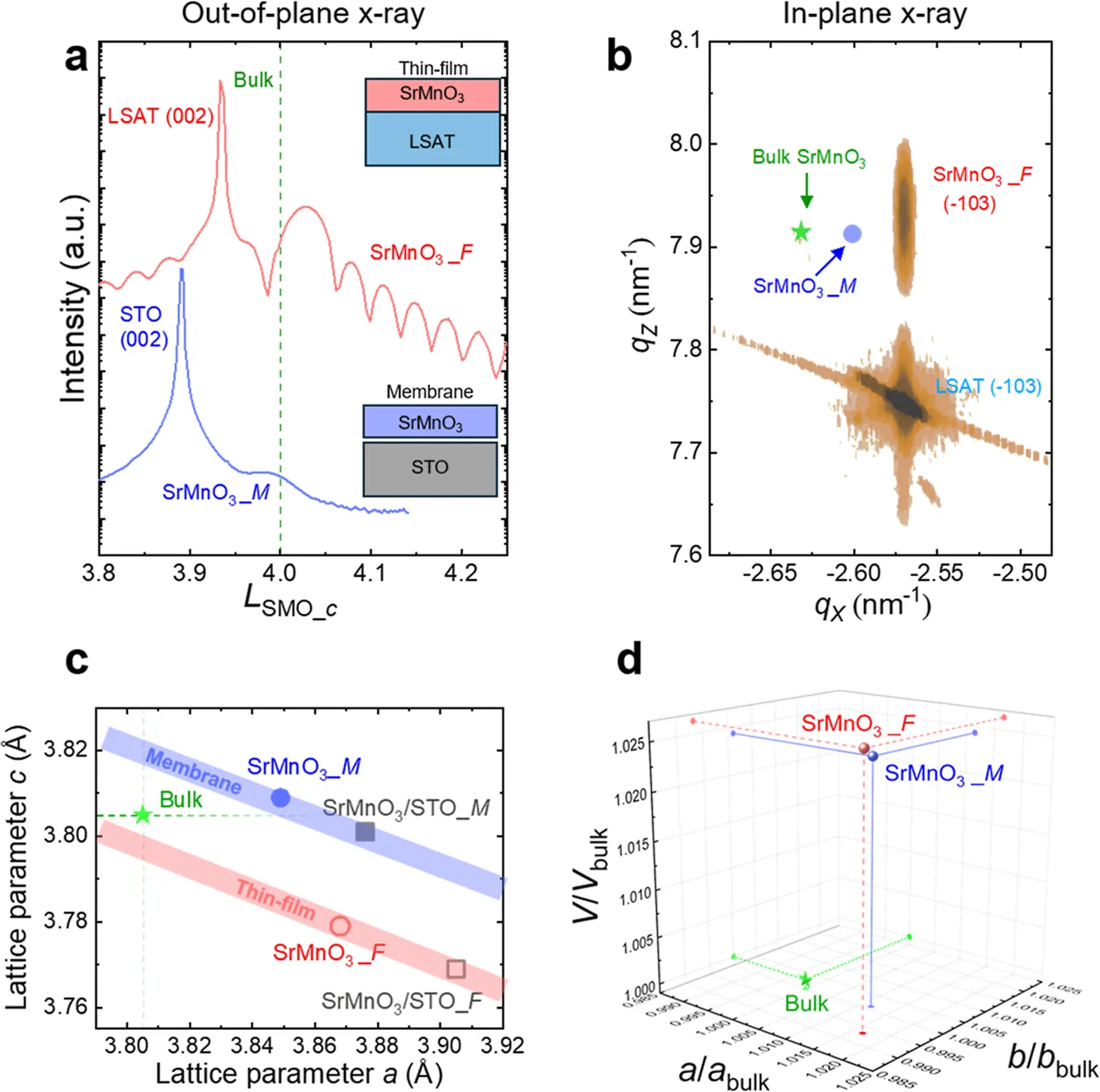
Determination of structural parameters through X-ray diffraction. (a) Synchrotron XRD patterns of the SMO/LSAT thin film (SrMnO_3__*F*) and corresponding freestanding membrane (SrMnO_3__*M*) sample around the (004) reflections. A clear shift of the membrane SMO peak toward bulk value indicates an increased lattice parameter after release. The green dashed lines indicate the bulk SMO lattice parameters. (b) Off-specular reciprocal space map (RSM) for the thin film (SrMnO_3__*F*) around the (−103) reflection, showing a coherent epitaxial growth. Line profiles were sliced at the center of the SMO and LSAT peaks. The calculated position of the membrane (SrMnO_3__*M*) from the derived lattice parameters (Figure S2) and bulk SMO are marked with a closed circle and star symbols, respectively. (c) In-plane “*a*” and out-of-plane “*c*” lattice parameters of the films and membranes derived from both LSAT and STO substrates. Thin film and membrane derived from STO substrates are denoted as SrMnO_3_/STO_*F* and SrMnO_3_/STO_*M* respectively. The green dashed lines indicate the bulk SMO lattice parameters, while the red and blue thick lines are guides to the eye highlighting the trends for the thin-film and membrane data, respectively. XRD data for the SMO/STO film and membrane are provided in Figure S2 of the Supporting Information (d) change in in-plane “*a*”, out-of-plane “*c*” lattice parameters and unit cell volume “*V*” relative to bulk SMO for the thin film and membrane samples, together with their projections onto the corresponding two-dimensional planes. Thin film unit-cell volume is larger than expected from conventional elastic strain accommodation based on typical oxide Poisson’s ratio and remains substantially expanded even after membrane release.

The intrinsic magnetic behavior of the films was verified through magnetization measurements, which show robust G-type antiferromagnetism with a Néel temperature *T*_N_ ≈ 269 K (Figure S6), consistent with phase-pure SrMnO_3_.^45^ Together, these measurements establish that the as-grown films are structurally coherent, chemically uniform, and magnetically intrinsic. Notably the Néel temperature remains close to the bulk value. As discussed below, this observation is consistent with the moderate oxygen-vacancy concentrations identified by X-ray photoelectron Spectroscopy (XPS) and density functional theory (DFT), which primarily manifest through lattice expansion without substantially altering the G-type antiferromagnetic ground state.^35^

Having established the high quality of the SrMnO_3_ thin films, freestanding membranes were fabricated. To do so, a polymer support layer (Methods and Figure S1) was used to hold the membrane during release from the substrate and during its transfer to the desired support (Figure 1c). We successfully fabricate large areas (5 × 5 mm^2^), crack- and wrinkle-free freestanding membranes (Figure 1d), ensuring reliable diffraction measurements and eliminating extrinsic strain from mechanical deformation.

### Irreversible Volume Expansion in the Thin-Film and Freestanding Membrane

To have a direct comparison of structural properties of the SrMnO_3_ thin films and freestanding membrane, synchrotron X-ray was employed for accurate out-of-plane and in-plane X-ray diffraction (XRD) (Figures 2 and S7). From the X-ray diffraction of the out of plane (004) peak (Figure 2a), partial strain relaxation is observed as the SrMnO_3_ peak shifts toward the bulk value. From the in-plane (−103) reflection of the thin film obtained by reciprocal space mapping (RSM) (Figure 2b) and the corresponding out-of-plane (004) and in-plane (100) reflections of the freestanding membrane measured by synchrotron X-ray diffraction (Figure S2), two further observations are obtained: (1) the thin film is highly coherent to the epitaxial substrate and (2) the membrane exhibits a clear shift of the in-plane lattice parameter toward the bulk value, indicating partial strain relaxation after lift-off. Similar data for the STO-based SrMnO_3_ film (SrMnO_3_/STO_*F*) and membrane (SrMnO_3_/STO_*M*) are shown in Figure S3, drawing identical observations. Additionally, the released membrane exhibits identical in-plane (100) and (010) lattice parameters (Figure S3c,d), confirming biaxial strain relaxation without measurable in-plane anisotropy.

From this systematic synchrotron based structural data, we deduced lattice parameters for both the thin films and corresponding membranes using Bragg’s law. The various thin film and membrane samples are described in Table 1, including detailed lattice parameters. For the thin-film (SrMnO_3__*F*), the measured lattice parameters (*c* = 3.779 Å, *a* = 3.868 Å) yield an in-plane tensile strain ε_*xx*_ = 1.66% and an out-of-plane strain ε_*zz*_ = −0.683%, which is consistent with earlier reports.^30,31,34^ Notably, the magnitude of ε_*zz*_ deviates from the elastic (Poisson) response (ε_*zz*_ ≈ −1.046%), resulting in a substantial unit-cell volume expansion of Δ*V*/*V* = 2.63%, as shown in Figure 2c,d.

**Table 1:** Details of Structural Parameters of SMO Thin Films and Membrane^a^

lattice parameters	film on LSAT (SrMnO_3__*F*)	film on STO (SrMnO_3_/STO_*F*)	membrane from LSAT (SrMnO_3__*M*)	membrane from STO (SrMnO_3_/STO_*M*)
	in-plane tensile strain ∼1.66%	in-plane tensile strain ∼2.63%		
*a* (Å)	3.868	3.905	3.849	3.873
*c* (Å)	3.779	3.769	3.809	3.801
*V* (Å^3^)	56.54	57.47	56.43	57.060
*V* (Å^3^) (Poisson’s ratio 0.3)	56.120	56.715	55.812	56.245

a The in-plane lattice parameter, *a*, and out-of plane lattice parameter, *c*, for the thin films and LSAT-based membrane. The observed sample volume and the expected volumes given the in-plane lattice and a typical perovskite ν = 0.3 is added for reference.

After substrate removal, membrane (SrMnO_3__*M*) exhibits lattice parameters *c* = 3.809 Å and *a* = 3.849 Å, corresponding to Δ*V*/*V* = 2.43% relative to bulk SrMnO_3_ (*a* = 3.805 Å), as shown in Figure 2c,d. Strikingly, the unit-cell volume that was expanded during SrMnO_3_ thin film growth on the LSAT substrate (imposing tensile strain) persists despite the absence of epitaxial clamping. A similar behavior was observed for freestanding membrane derived from STO substrate (SrMnO_3_/STO_*M*). Notably, the 2 nm STO barrier layer is unlikely to be responsible for this volume expansion, as comparable lattice expansion is observed in membranes released from STO substrates where the STO layer is homoepitaxial and does not introduce additional lattice-mismatch strain. If the unit cell volume expansion in the thin films were purely elastic (epitaxial strain driven), full relaxation toward the bulk lattice would be expected upon release from the substrate. The persistence of such a large volume expansion therefore points to an intrinsic structural modification beyond conventional elastic strain relaxation.

Previous studies have shown that tensile strain in SrMnO_3_ promotes oxygen-vacancy formation, resulting in lattice expansion and significant modifications of its electronic and magnetic properties.^38–40,46^ In conventional substrate-supported films, however, these defect-mediated structural changes remain coupled to the applied epitaxial strain. By contrast, the freestanding membrane geometry enables direct separation of elastic and defect-driven contributions. To further probe the role of oxygen vacancies, we performed postrelease oxygen annealing on the freestanding membranes (Figure S4). Oxygen annealing progressively reduced the out-of-plane lattice parameter toward the bulk value, consistent with partial removal of oxygen vacancies. However, even after annealing at 600 °C,^7^ the lattice parameter remained measurably larger than the bulk value, indicating that oxygen stoichiometry alone cannot fully account for the persistent lattice expansion. Together, these results suggest that the retained volume expansion originates from the combined effects of oxygen-vacancy-induced lattice expansion and a persistent epitaxial structural memory inherited from epitaxial growth. Understanding the origin of this persistent volume expansion is important for designing oxide heterostructures and functional devices that exploit strain-defect coupling to stabilize nonequilibrium states.

## Origin of Irreversible Volume Expansion

### X-ray Photoelectron Spectroscopy

To investigate the origin of the persistent unit cell volume expansion in substrate released freestanding membrane, oxygen-vacancies were considered. Tensile strain in perovskite oxides is known to reduce their formation energy by accommodating lattice expansion,^11,16,24,34^ and so are expected in large numbers in the tensile-strained samples. To directly assess whether such oxygen vacancies are present in our samples, we quantified the Mn-oxidation state, which is directly impacted by the oxygen vacancy, lowering the ideal Mn^4+^ valence state to balance the electronic charges.^35–37^ We study Mn 3*s* multiplet splitting in X-ray photoelectron spectroscopy (XPS) (Figure 3a), which was used to quantify the valence state.^47–50^ Spectra for the films and membranes were collected alongside MnO_2_ (reference for Mn^4+^ state) and Mn_2_O_3_ (reference for Mn^3+^ state) powders (Figure 3b). To extract accurate splitting energies, we fit the spectra using a simple Gaussian peak function, well capturing the peak position and estimated the energy separation (Δ*E*) between two peak positions as shown in Figure 3c, which reveal a systematic reduction in Mn valence toward more Mn^3+^ states, indicating more oxygen vacancies, with increasing tensile strain (Figure 3d).^30,49,50^

**Figure 3:**
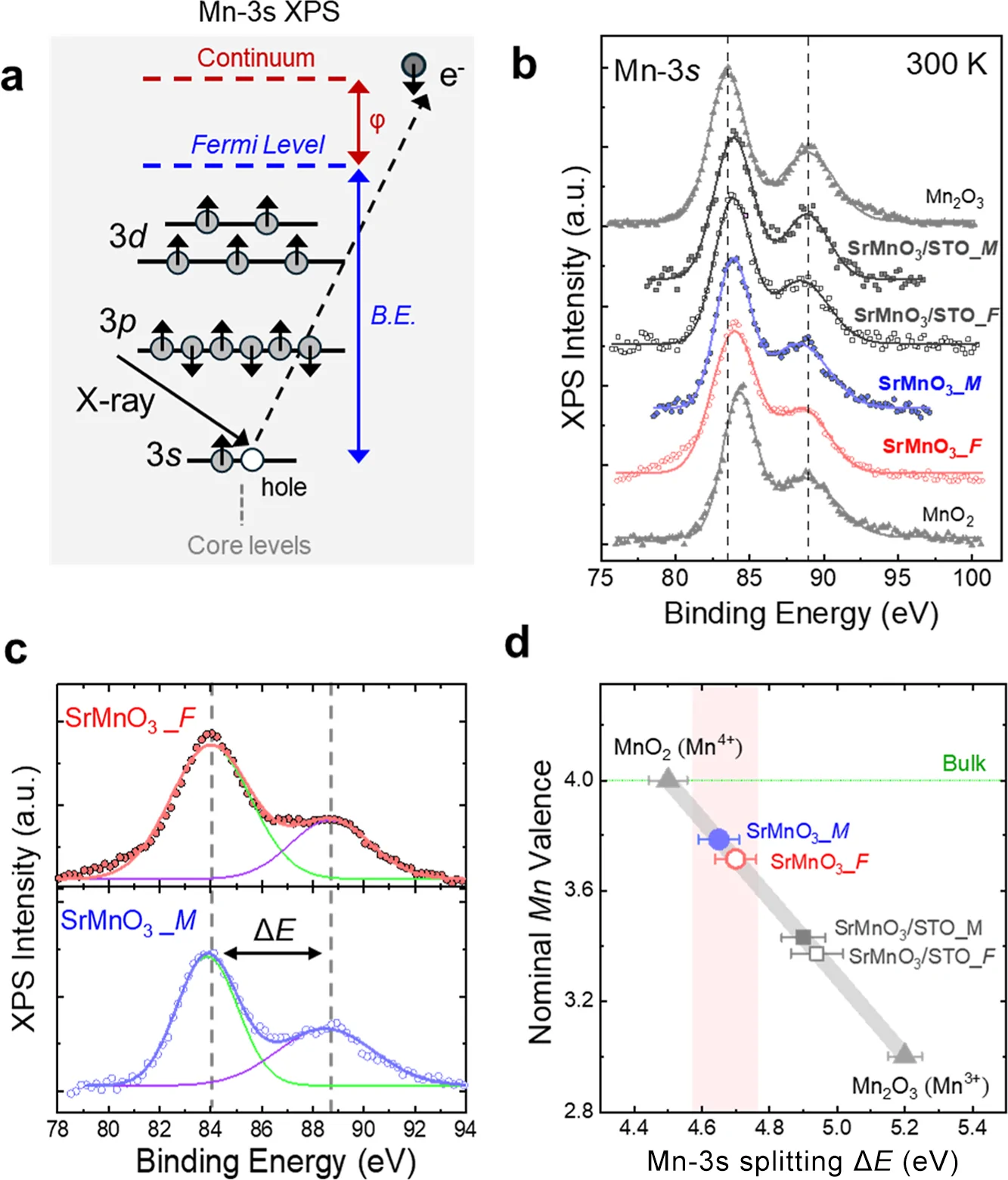
X-ray photoelectron spectroscopy studies of the Mn-oxidation state. (a) Schematic of the level schemes for the Mn 3s XPS spectra. (b) Mn-3s XPS spectra from various samples and reference MnO_2_ and Mn_2_O_3_ powders. (c) A representative Mn-3s XPS spectra fitting of the thin-film and membrane samples. A near-identical peak separation can be seen. (d) Oxidation states for reference powders, thin films, and membranes. The solid line is a guide to the eye. Membranes and films show almost identical oxidation states for the same growth substrate. Also, with increasing tensile strain, Mn-oxidation is more toward Mn^3+^ state, indicating more oxygen vacancies.

In particular, the film grown on STO (SrMnO_3_/STO_*F*), which experiences larger tensile strain, exhibits a lower Mn-oxidation state than that of the thin film grown on LSAT with reduced tensile strain. This indicates the presence of more oxygen vacancies in SrMnO_3_ films that have grown at higher tensile strain state. Importantly, the corresponding membranes display oxidation states within experimental uncertainty of their parent films, indicating that oxygen vacancy concentrations remain largely unchanged upon exfoliation (Figure 3c,d). The average Mn valence obtained from XPS measurements corresponds to an oxygen-deficient composition of approximately SrMnO_2.88_ (δ ≈ 0.12) for the freestanding membrane grown on LSAT, which falls well within the range reported for bulk SrMnO_3‑δ_ (δ ≈ 0.1–0.18).^30,39,41,46^ Although XPS is inherently surface sensitive,^51^ the present SMO films are only ∼11 nm thick and remain below the critical thickness (∼15–20 nm) for misfit-dislocation formation reported in tensile-strained SMO films.^30^ Consequently, the films are expected to remain coherently strained and structurally homogeneous throughout their thickness. Under such conditions, significant depth-dependent variations in oxygen-vacancy concentration are not anticipated between the surface and bulk regions. Therefore, the Mn valence measured by XPS is expected to be broadly representative of the entire film and freestanding membrane thickness. These XPS results demonstrate that strain-induced oxygen vacancies incorporated during high-temperature growth are largely retained after substrate removal. Combined with the oxygen annealing results (Figure S4), they indicate that oxygen vacancies make a substantial contribution to the lattice expansion, while the incomplete recovery after oxygen annealing reveals an additional contribution from persistent epitaxial structural memory.

### Density-Functional Theory

To understand the thermodynamic origin of this increased defect concentration, density-functional theory calculations^31^ with Hubbard corrections (Methods) were performed to evaluate oxygen-vacancy formation energies as a function of biaxial strain and temperature.^46,52,53^ The calculations show that tensile strain significantly lowers the vacancy formation energy,^16,53^ particularly under typical growth temperatures (∼1013 K) (Figure 4a), which is in excellent agreement with the XPS measurements. Under these conditions, the predicted equilibrium vacancy concentration exceeds 1% per oxygen site at tensile strain levels comparable to experimental levels (Figure 4b). These results establish that oxygen vacancies are thermodynamically favored during tensile-strained film growth and may become kinetically frozen upon cooling.

**Figure 4:**
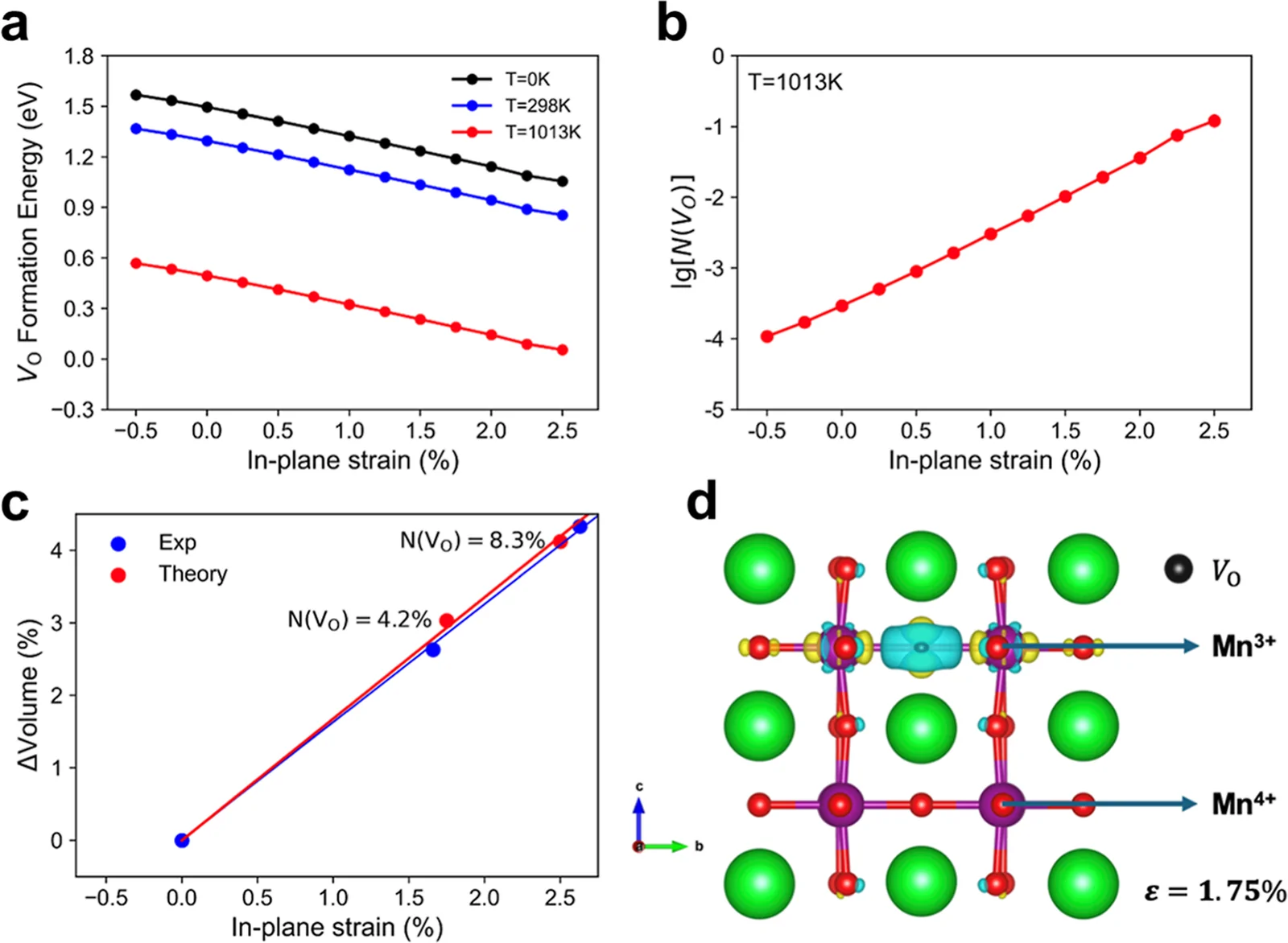
First-principles calculations revealing mechanism of volume expansion under tensile strain. (a) Biaxial strain dependence of the oxygen vacancy formation energy in SMO at different temperatures. As synthesis T and strain ε increase, oxygen vacancy formation energy lowers. (b) Effect of strain on oxygen vacancy concentration at 1013 K derived from the formation energy calculated in (a). (c) Comparison of the experimental SMO volume change with theoretical DFT results with increasing strain. The corresponding oxygen vacancy concentration at different strain is obtained from (b) for theoretical results. The volume change of the SMO/LSAT film referenced to pristine closes matches under a *V*_O_ of 4.2%, with an increased concentration of 8.3% matching the SMO/STO film. The slope for volume change as a function of strain is in good agreement between theory and experiments. (d) Differential charge density Δρ = ρ_defect_ – (ρ_pristine_ – ρ_O_) of an SMO film with an oxygen vacancy *V*_O_ under 1.75% epitaxial strain. Electron density isosurface is 0.01 e/Å^3^, yellow indicates an increase in charge density, while blue-green indicates a decrease in charge density.

Next, the effect of these frozen vacancies on the crystal structure was examined. Structural relaxations performed at experimentally relevant strain states reproduce the observed excess unit-cell volume (Figure 4c). Microscopically, oxygen vacancy formation induces the reduction of two neighboring Mn^4+^ ions to Mn^3+^, consistent with our direct experimental observation in XPS. Bader charge analysis indicates an electron gain of ∼0.29 e on the Mn ions adjacent to the vacancy, while more distant sites remain essentially unchanged (<0.02 e), confirming localized reduction. Because Mn^3+^ possesses a larger ionic radius and weaker Mn 3d-O-2p hybridization, the Mn^3+^–O–Mn^4+^ bond angle decreases from 180° to ∼171°, accompanied by a Mn–Mn distance increase of ∼0.011 Å. This local structural relaxation produces a net expansion of the unit cell.

Taken together, experimental and theoretical results establish a consistent mechanism. Tensile strain lowers the oxygen-vacancy formation energy during growth, generating defect concentrations sufficient to expand the lattice volume. Once incorporated, these vacancies remain stabilized and do not annihilate upon substrate removal, leading to the observed irreversible volume expansion in freestanding membranes.

## Conclusion and Outlook

In summary, we have demonstrated that epitaxial tensile strain in SrMnO_3_ epitaxial thin films induces a substantial unit-cell volume expansion that remains kinetically frozen even after the fabrication of freestanding membranes. Synchrotron X-ray diffraction provides direct evidence that the lattice does not relax toward the bulk structure upon substrate removal, marking a clear departure from purely elastic behavior. Our combined XPS and first-principles calculations establish that this “frozen epitaxial strain” is fundamentally chemical in origin: tensile strain lowers the oxygen-vacancy formation energy during growth, leading to Mn reduction and a defect-stabilized lattice that resists relaxation.

These findings reveal that epitaxial strain in complex oxide thin films is an irreversible process governed by strain-driven defect thermodynamics rather than simple mechanical deformation. This necessitates a new framework for interpreting strain-property relationships—one that disentangles purely elastic effects from persistent chemical modifications. More broadly, this work establishes freestanding membranes as a powerful diagnostic platform for probing irreversible strain accommodation in functional oxides. By extending this approach to other perovskite systems, correlated oxides, and multiferroics, researchers can finally clarify the long-standing discrepancies between predicted intrinsic phases and experimentally realized behavior in strain-engineered heterostructures.

## Methods

### Thin Film Growth

SrMnO_3_ (SMO) (00L) thin films were deposited by pulsed laser deposition using an excimer laser with a wavelength of 248 nm on (001)-oriented substrates. During the growth, the pressure was kept at 10 mTorr of oxygen with a temperature of 740 °C. A laser energy density of 1.5 J/cm^2^ with a frequency of 3 Hz was employed to grow 11 nm films. For the membranes, a heterostructure consisting of 11.5 nm SMO, a 2 nm SrTiO_3_ diffusion barrier, and 11 nm of SrCa_2_Al_2_O_6_ (SCAO) as a sacrificial layer was grown on the SrTiO_3_ substrate. SCAO growth was done at 7.5 mTorr and 780 °C, with a laser density of 1 J/cm^2^ and frequency of 5 Hz. The STO was deposited at 75 mTorr, 780 °C, 1.12 J/cm^2^ and 5 Hz. All films were cooled down in about 260 Torr oxygen after growth.

### Membrane Fabrication

Membrane fabrication was done using a polymer-supported technique (Figure S1). The thin-film heterostructures were first cleaned with acetone and IPA before Poly­(methyl methacrylate) (PMMA) and Polypropylene carbonate (PPC) were sequentially spin-coated onto the film. First PMMA layer was spin coated at 5000 rpm for 1 min which was followed by a baking at 180 °C for 5 min. Then a PPC layer was coated at 1000 rpm for 1 min, which was followed by a baking at 45 °C for 60 min. The sample was then floated on water to etch the SCAO sacrificial layer. After etching, the membrane was collected and transferred onto an STO substrate for support. The polymers were then removed via dissolution in acetone to yield the large-scale freestanding membranes.

### Magnetic Measurement

The temperature-dependent magnetism for the SrMnO_3_ thin film on LSAT was measured using a SQUID magnetometer in the out-of-plane [00L] direction. Both zero-field cooled (ZFC) and field cooled (FC) curves were collected from 10 to 385 K (Figure S6). The magnetic field was set at 20 kOe during cooling for the FC curve, and 0.1 T during the measurements. The LSAT substrate was similarly measured in isolation and used to correct for substrate background signals.

### X-ray Diffraction

A lab-source X-ray diffractometer (Bruker D8 Discover) was employed to perform nonspecular reciprocal space mapping (RSM) measurements around the (−103) Bragg peak to verify the fully epitaxial strain state of the SrMnO_3_ thin films grown on respective substrates. The precise lattice parameters of the SrMnO_3_ thin films and released membranes were determined by synchrotron X-ray diffraction measurements using a six-circle Huber diffractometer configured with Psi-C geometry at the beamline 7-ID-C of the Advanced Photon Source at Argonne National Laboratory. A Si(111) double-crystal monochromator with an energy resolution of Δ*E*/*E* = 1 × 10^–4^ was employed to fine-tune the X-ray energy at 17.5 keV and 18.3 keV (wavelength λ = 0.70846 Å and 0.67749 Å). The X-ray beam at 7-ID-C with a total flux of 3 × 10^12^ photons per second was optically focused by using Kirkpatrick-Baez (K–B) mirrors, resulting in a beam profile of 30 μm (Vertical) × 50 μm (Horizontal). A small vertical beam profile is optimal for the grazing incidence in-plane scattering. Scattering signals were recorded using an Eiger2 × 500 K 2D area detector, and the 2D images were subsequently processed to obtain the line profiles of diffraction peaks to determine the lattice parameters. X-ray diffraction measurement geometries (e.g., both out-of-plane specular and grazing incidence in-plane diffraction) for the epitaxial thin film and membrane are given in Figure S7. Lab-source XRD of the heterostructures prior to exfoliation is presented in Figure S8.

### X-ray Photoelectron Spectroscopy

X-ray photoelectron spectroscopy (XPS) measurements were performed using a Thermo Scientific K-Alpha system with a pass energy of 50 eV, a spot size of 400 μm, and a step size of 0.2 eV. A total of 200 scans were acquired and averaged to improve the signal-to-noise ratio. The Mn-oxidation state was determined from the Mn 3s multiplet splitting. Charging effects in both thin films and membranes were corrected by referencing the C 1s peak.

### High-Temperature Annealing under Oxygen Gas Flow

Freestanding SMO (10 nm)/STO (2 nm) membranes were transferred onto STO (001) substrates with a 4° miscut along the [110] direction. The as-transferred membranes were first immersed in acetone to remove the polymer support layer. Each membrane was then cut into two pieces of equal size, with one piece annealed in an oxygen atmosphere at a temperature of 250 or 600 °C in a tube furnace for 2 h. Structural characterization of the as-transferred, 250 °C-annealed, and 600 °C-annealed SMO membranes was performed using XRD (Bruker D8 Discover) using Cu Kα radiation (λ = 1.5418 Å).

### DFT Calculations

The calculations were performed with density functional theory (DFT) in the Vienna ab initio simulation package (VASP),^54,55^ utilizing the projected augmented wave (PAW) pseudopotentials^56^ and Perdew–Burke–Ernzerhof (PBE) exchange–correlation functionals.^57^ To address strong correlation effects, we add the Hubbard correction PBE + U method^58^ in a rotationally invariant formalism^59^ with *U* = 3.0 eV for the Mn-3d orbitals, as has been used in previous studies of SrMnO_3_.^34,41,60^

We studied the G-type antiferromagnetic ground state of SMO.^60^ The magnetic primitive cell ( $\sqrt{2}\times \sqrt{2}\times 2$ cubic primitive cell) was fully relaxed at zero strain with a force convergence criterion of 0.01 eV/Å and used as the reference structure. To simulate the experimental strain conditions, biaxial strain was applied by fixing the in-plane lattice constants at values obtained by straining the reference structure to each experimental strain state, while the out-of-plane lattice constant and all atomic positions were relaxed. This constrained cell relaxation is not natively supported in VASP and was implemented through a modification of the source code.

For the calculation of defect formation energies, we employed a 160-atom $2\sqrt{2}\times 2\sqrt{2}\times 4$ cubic primitive cell to ensure isolated defects. The defect-containing supercells were relaxed at a fixed volume until the forces on each atom were less than 0.02 eV/Å. The oxygen vacancy was placed in the MnO_2_ (001) plane, as its formation energy is lower than that in the SrO (001) plane. At 1.75% tensile strain, for example, the formation energy in the MnO_2_ plane is reduced by about 0.20 eV compared to the SrO plane. This energy difference becomes larger with increasing strain, highlighting the growing preference for vacancy formation in the MnO_2_ plane. To investigate the effect of high oxygen vacancy concentration on volume changes, we used $\sqrt{2}\times \sqrt{2}\times 2$ and 2 × 2 × 2 supercells, removing one oxygen atom and relaxing the structure under the same fixed in-plane lattice parameters. The force convergence criterion was set to 0.01 eV/Å.

### Defect Formation Energy and Concentration Calculations

Given that experimental measurement shows this sample is insulating with minimum carriers, we consider only neutral oxygen vacancy in this study. The formation energy of a charge-neutral oxygen vacancy is expressed as

$$\Delta H_{f}\left(V_{O}\right)=E\left(d\right)-E_{h}+\mu_{O}$$

where *E*(d) and *E*_h_ are the total energies of the systems with and without the defect, and μ_O_ is the oxygen chemical potential. We consider the effect of temperature on the oxygen chemical potential based on the following equation.^41,45,60^

$$\mu_{O}=\frac{1}{2}\left[\Delta H-T\Delta S+\mu_{O_{2}}\left(p^{O},0K\right)\right]$$

Δ*H* and Δ*S* for O_2_ gas can be obtained from ref 60. μ_O2_ (*p* ^O^, 0 K) is the chemical potential of the O_2_ molecule from DFT total energy with a correction of 5.1 eV to the binding energy according to experimental value.^60^ At a given temperature T, the equilibrium oxygen vacancy concentration is evaluated as^41,60^

$$N\left(V_{O}\right)=N_{A}\exp\left(-\Delta H_{f}/k_{B}T\right)$$

where the *N*_A_ is the number of equivalent oxygen sites in the unit-cell.

## Supplementary Material



## Notes

Supporting figures and texts on freestanding membrane fabrications; additional X-ray characterizations; atomic force microscopy images, and magnetic measurements (PDF)
