# Supplementary material for: Frozen Epitaxial Strain in Complex Oxide Freestanding Membranes
Source: ACS Nano. 2026 Oct 5;20(41):27728–37. doi: 10.1021/acsnano.6c08965 (PMC13689926; doi:10.1021/acsnano.6c08965)
Supplement: Supplementary file 1 [file nn6c08965_si_001.pdf]

Supplemental Information for

Frozen epitaxial strain in Complex Oxide Freestanding Membranes

Paul Lenharth,[1,†] Yuchuan Yao,[1,†] Shihao Tu,[1] Pratap Pal,[1,*] Varun Nitin Mapara,[2] Ruiqi Sun,[1] Wuzhang Fang,[1] Peyton Burden,[3] Tula R. Paudel,[3] Jason Kawasaki,[1] Uwe Bergmann,[2] Hua Zhou,[4] Yuan Ping,[1] and Chang-Beom Eom[1,*]

[1]Department of Materials Science and Engineering, University of Wisconsin-Madison, Wisconsin 53706, USA

[2]Department of Physics, University of Wisconsin–Madison, Madison, Wisconsin 53706, USA

[3]Department of Physics, South Dakota School of Mines and Technology, Rapid City, South Dakota 57701, USA

[4]X-ray Science Division, Advanced Photon Source, Argonne National Laboratory, Lemont, Illinois 60439, USA

* Corresponding authors: ppal3@wisc.edu, ceom@wisc.edu

† These authors contributed equally

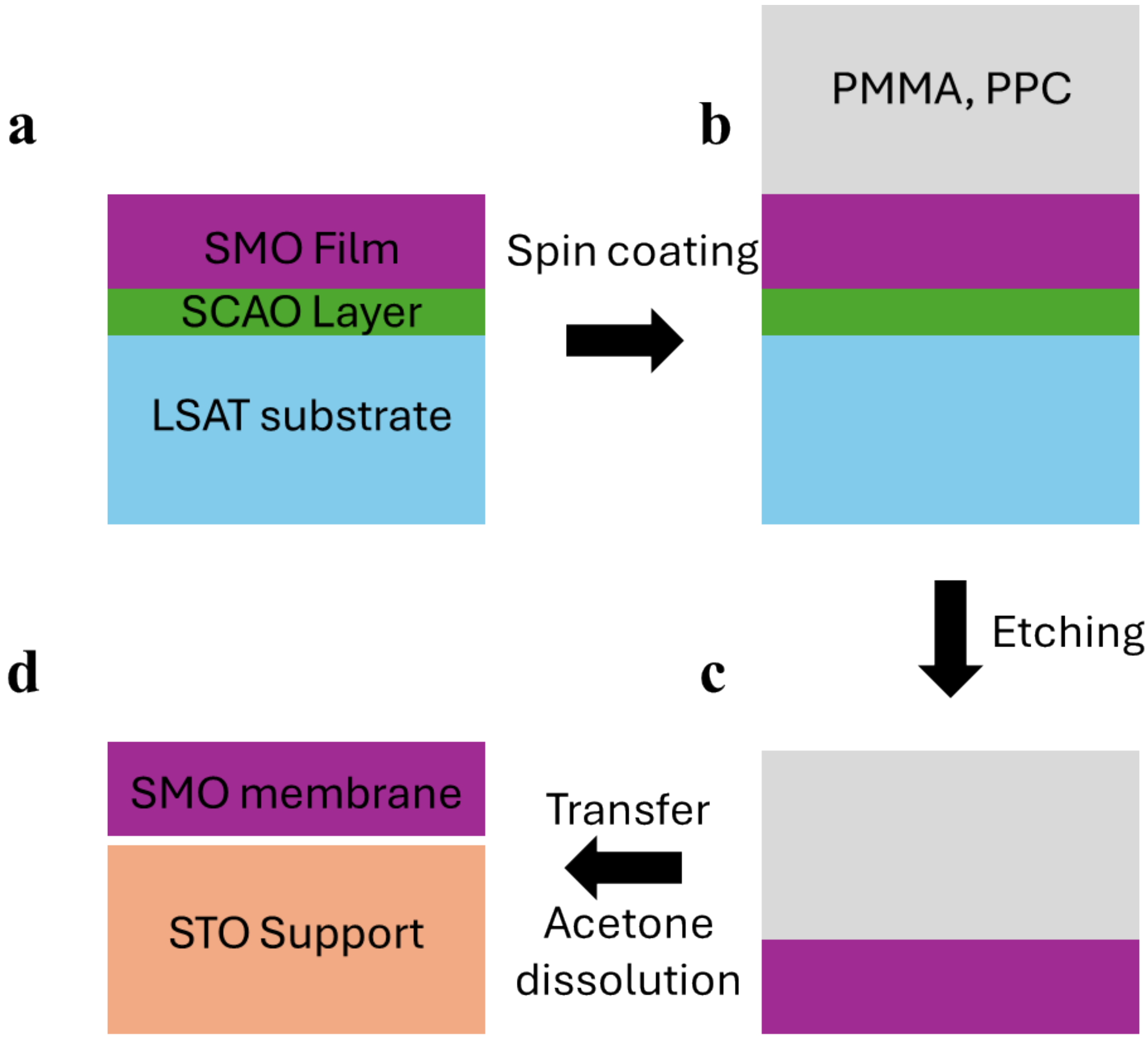

Fig. S1: **Membrane fabrication process:** (a) SMO/SCAO/LSAT heterostructure growth by PLD. (b) Application of a PMMA/PPC polymer support layer to the SMO film during selective etching of SCAO layer. (c) Removal of the SCAO sacrificial layer by water etching, resulting in a freestanding SMO membrane supported by the polymer layer. (d) Transfer of the membrane onto an STO support followed by removal of the polymer support using acetone and subsequent rinse with IPA.

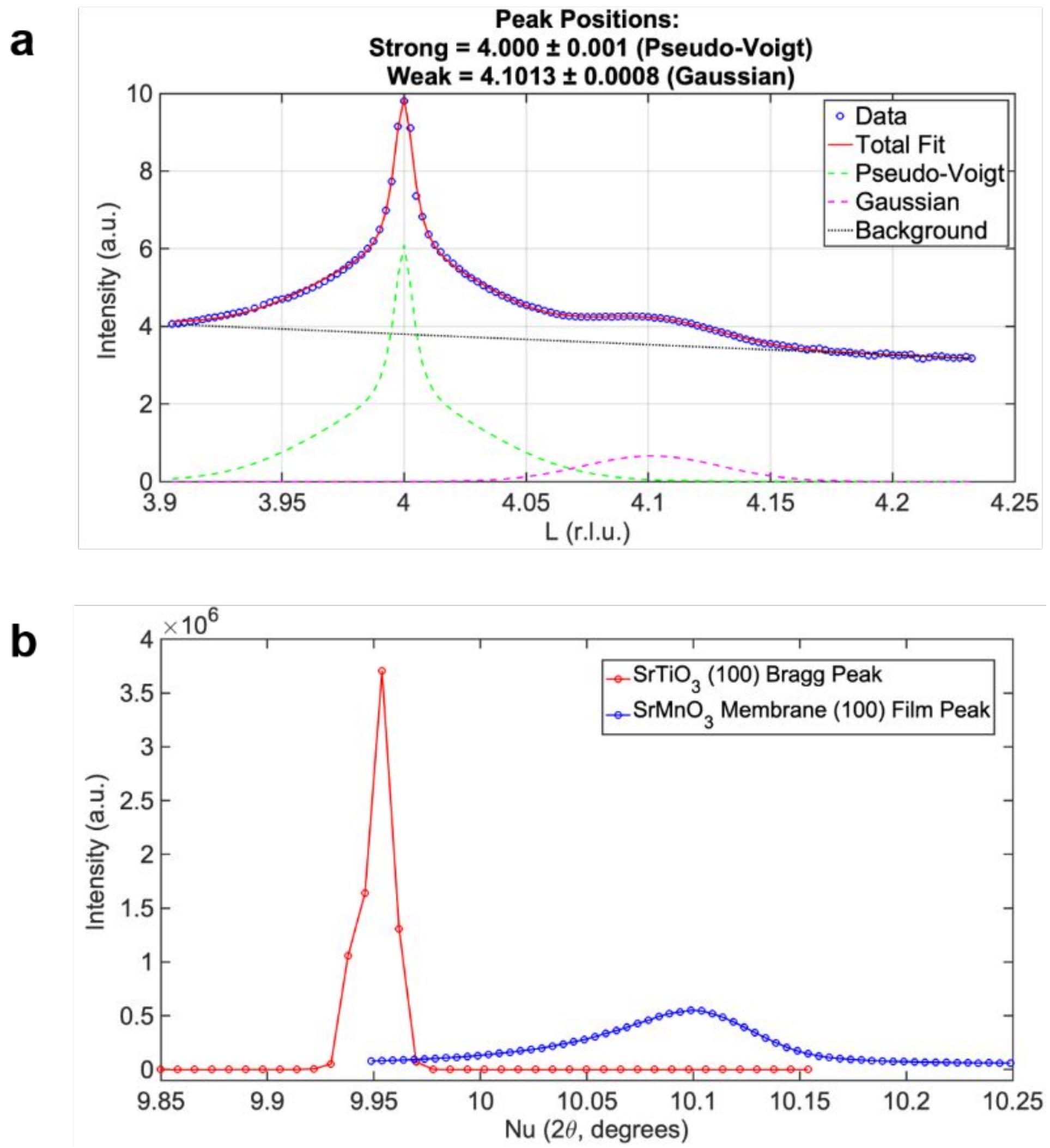

Fig. S2: **Lattice parameter characterization of the freestanding $SrMnO_3$ membrane**. (a) Out of plane (004) peak and (b) In plane grazing incidence (100) peak of the freestanding SMO membrane derived from LSAT substrate and collected on $SrTiO_3$ substrate, as measured by synchrotron X-ray source (X-ray energy 18.3 kV).

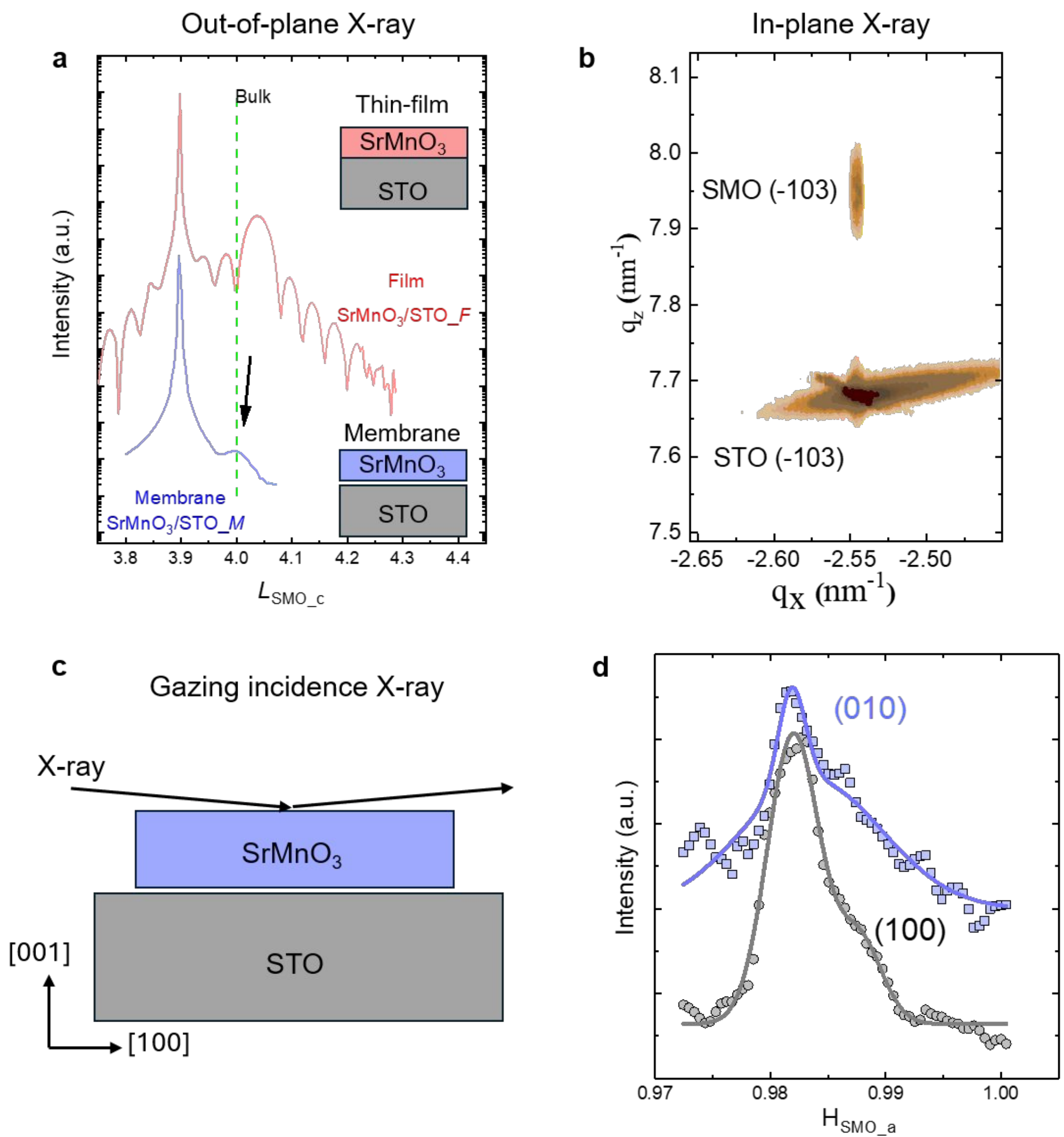

Fig. S3: **XRD of the $SrMnO_3$ thin film and membrane derived from STO substrates.** (a) Out-of-plane specular diffraction of the SMO thin film and membrane derived from STO substrate. Upon fabrication a clear relaxation can be seen. (b) RSM spectrum of the SMO/STO epitaxial thin film, showing a coherent epitaxial growth. (c) Schematic illustration of Grazing incidence in-plane X-ray diffraction. (d) Corresponding spectra of (100) and (010) peaks of the SMO membrane produced from a STO-based heterostructure and transferred on another STO substrate.

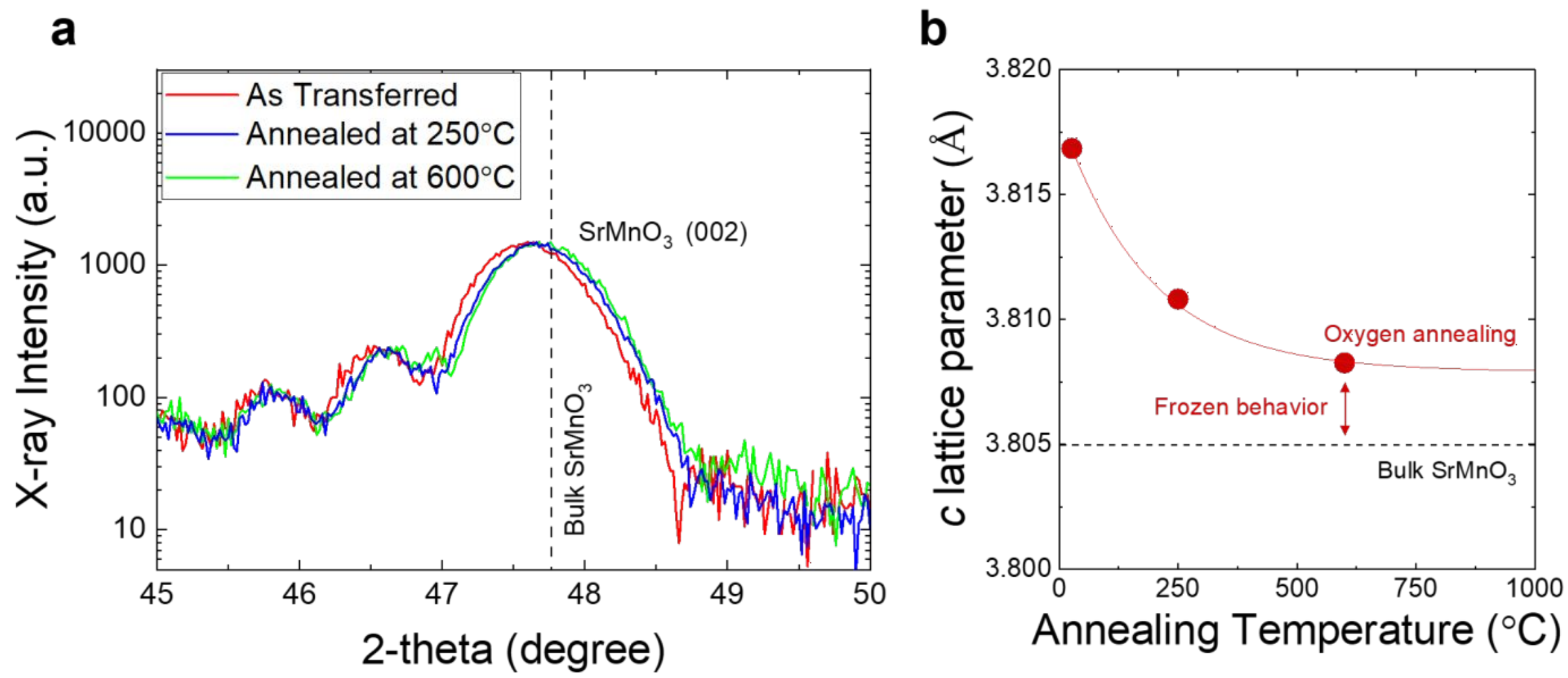

Fig. S4: **Effect of post-release oxygen annealing on the lattice parameter of freestanding $SrMnO_3$ membranes. (a)** Out-of-plane X-ray diffraction (002) scans of the freestanding $SrMnO_3$ membrane in the as-transferred state and after annealing under flowing oxygen at 250 °C and 600 °C, showing a systematic shift of the $SrMnO_3$ (002) peak toward higher 2θ with increasing annealing temperature. **(b)** Corresponding out-of-plane lattice parameter extracted from the diffraction peak position. Although oxygen annealing progressively reduces the lattice parameter toward the bulk $SrMnO_3$ value, it does not fully recover even after annealing at 600 °C, suggesting that, in addition to oxygen stoichiometry, persistent structural effects inherited from epitaxial growth contribute to the residual lattice expansion.

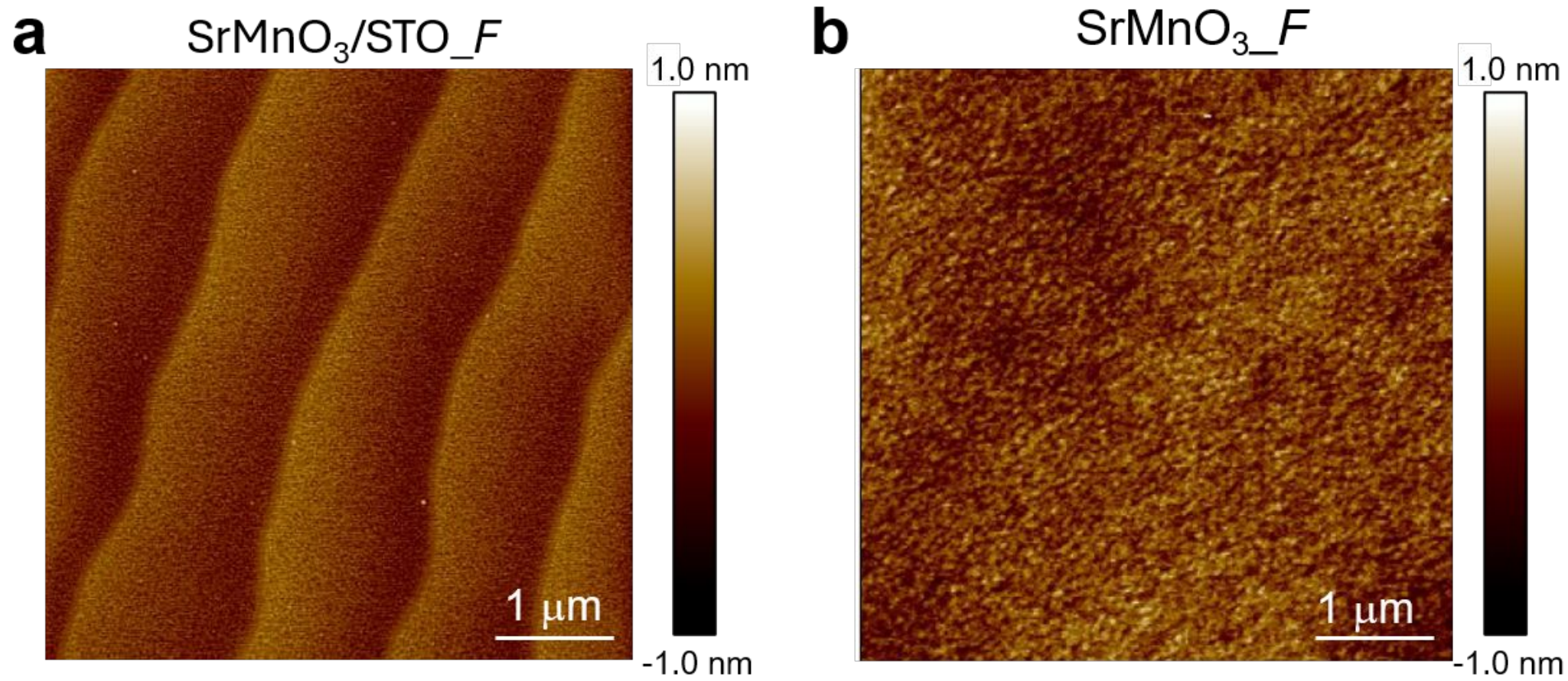

Fig. S5: **AFM of the Thin films and Heterostructures.** The AFM images of (a) SMO/STO thin film, (b) SMO/LSAT thin film. All films are atomically smooth with RMS roughness below 0.3 nm.

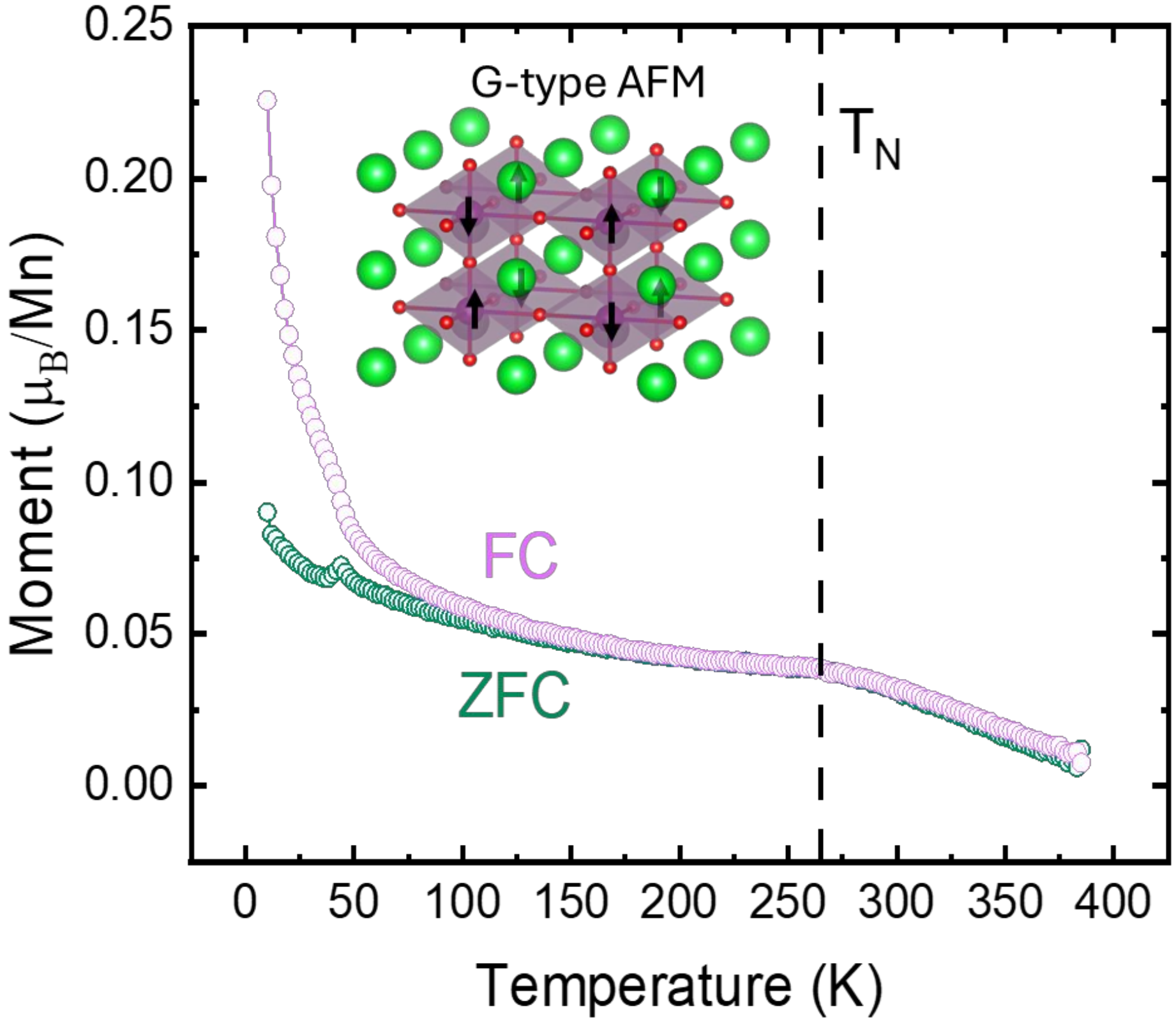

Fig. S6: **Magnetism of the thin film.** Temperature dependent magnetization measurement of the SMO/LSAT (001) thin film using SQUID magnetometry. A robust antiferromagnetic signal is evident. The Néel temperature, 269 K, is marked by the dashed line.

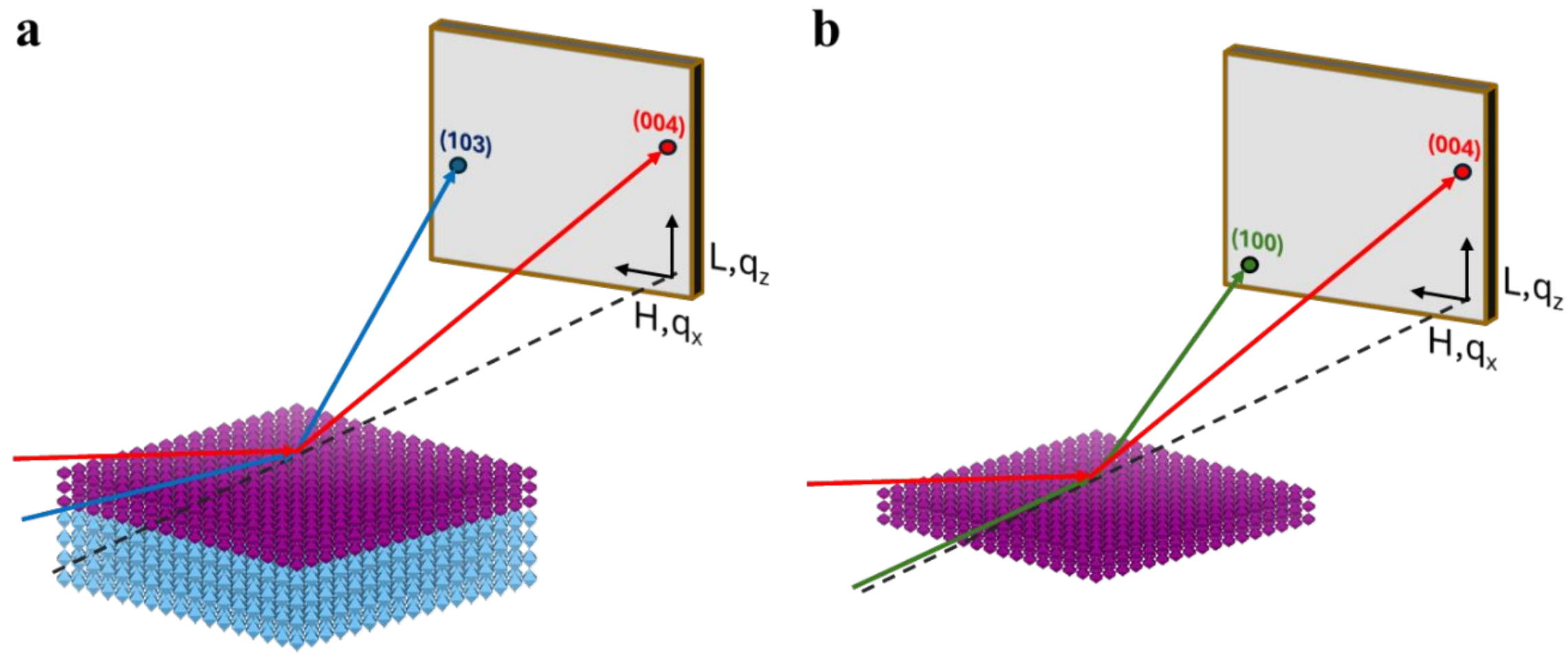

Fig. S7: **X-ray Diffraction Schematics.** XRD diffraction geometries for (a_ the thin film samples and (b) the membrane samples.

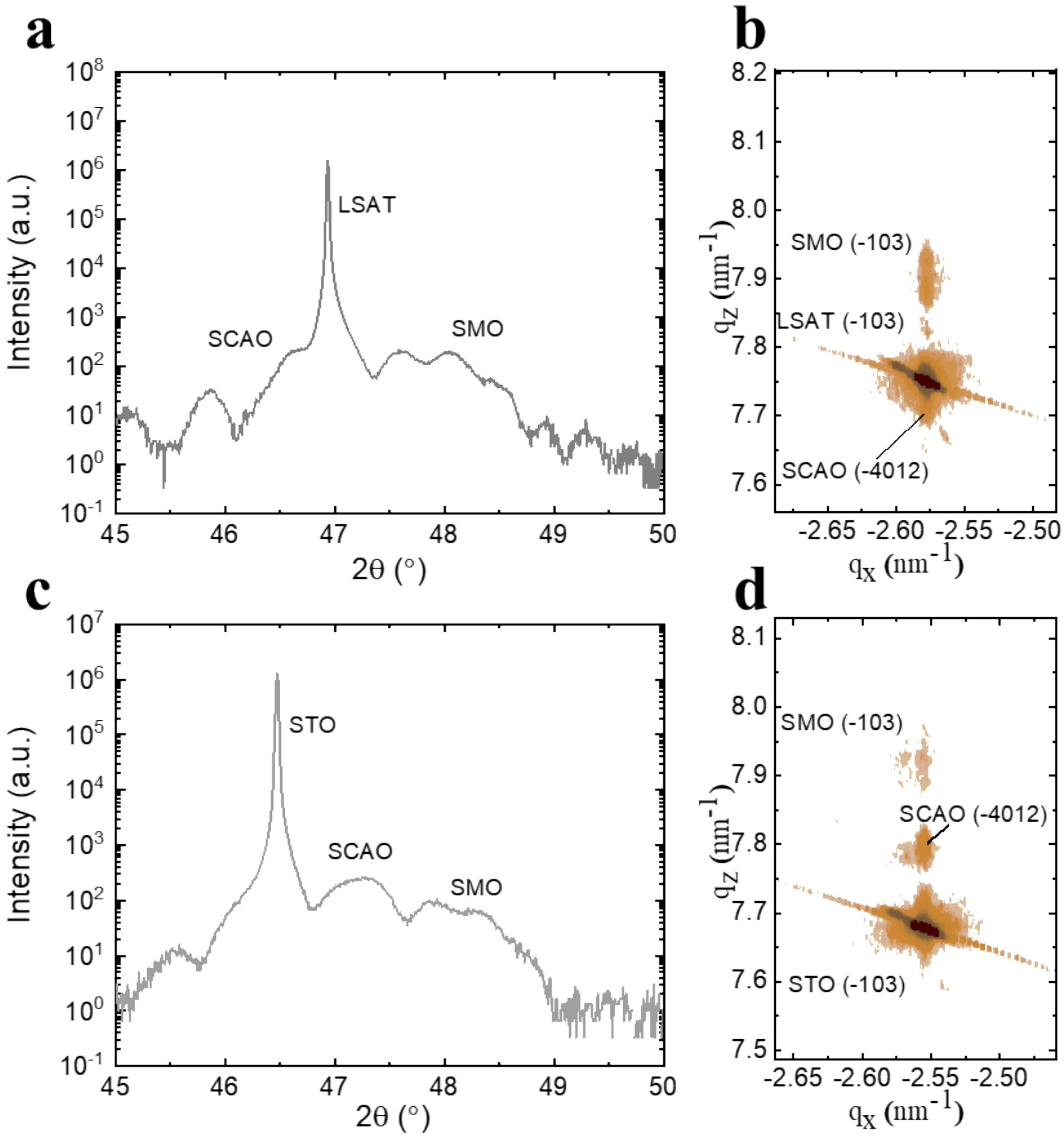

Fig. S8: **XRD of the Heterostructures.** (a) Lab-source specular diffraction scan of the SMO/STO/SCAO/LSAT (001) heterostructure used to fabricate the LSAT-based membrane. (b) RSM around the (-103) peak of the LSAT heterostructure. Alignment of the peaks in q_x reveals a coherent growth. (c) (002) scan for the heterostructure on STO. (d) (-103) RSM of the STO heterostructure. A slight relaxation is observed, likely due to the larger tensile strain on STO compared to LSAT for the SCAO and SMO layers.
